# Supplementary material for: Single-Shot Multi-Frame Imaging of Femtosecond Laser-Induced Plasma Propagation
Source: Materials (Basel). 2023 Apr 21;16(8):3264. doi: 10.3390/ma16083264 (PMC10142422; doi:10.3390/ma16083264)
Supplement: Supplementary file 1 [file materials-16-03264-s001.zip › materials-2290409-supplementary.pdf]

Article

Single-shot multi-frame imaging of femtosecond laser-induced plasma propagation

Tianyong Zhang [1], Baoshan Guo [1,2], Lan Jiang [1,2,3] *, Tong Zhu [1], Yanhong Hua [1], Ningwei Zhan [1] and Huan Yao [1]

[1] Laser Micro/Nano Fabrication Laboratory, School of Mechanical Engineering, Beijing Institute of Technology, Beijing, 100081, China
[2] Yangtze Delta Region Academy of Beijing Institute of Technology, Jiaxing, 314000 Zhejiang, China;
[3] Beijing Institute of Technology Chongqing Innovation Center, Chongqing, 401120, China;
* Correspondence: jianglan@bit.edu.cn

1. Generation of the sub-pulses and definition of the 0.0 ps with the pump pulse

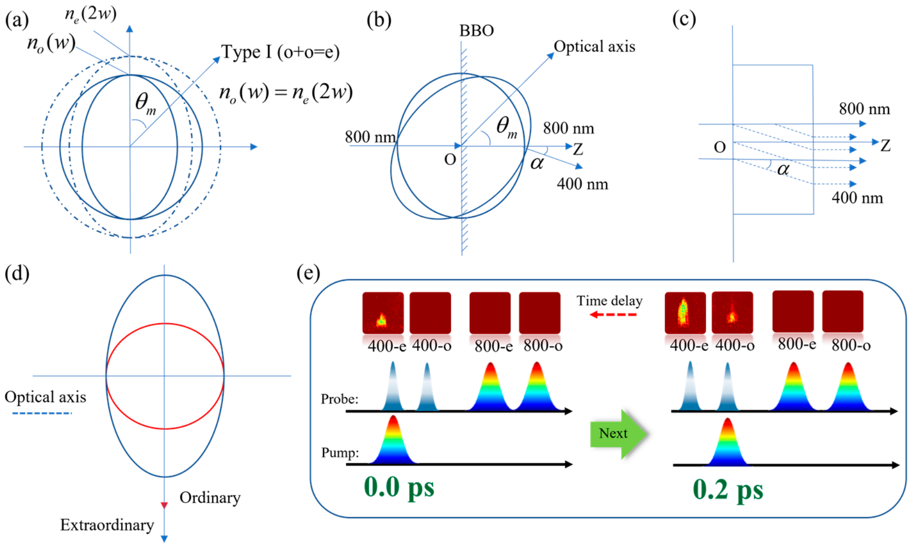

Figure S1. Principle of multi frequency and polarization pulse generation. (a) Phase matching method of negative uniaxial crystal; (b) Phase matching angle of the BBO crystal; (c) Spatial walk-off angle of BBO crystals; (d) Birefringence of quartz crystals; (e) The time delay definition of pump and probe sub-pulses.

2. Spatial resolution test

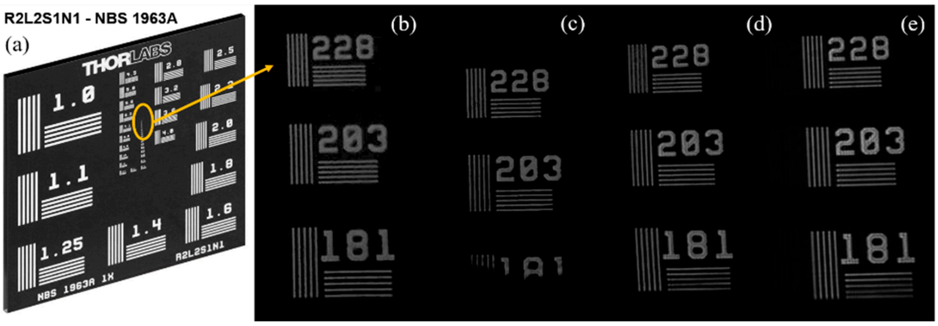

Figure S2. Spatial resolution test. (a) Standard resolution test target, maximum 228 cycles/mm; (b-e) Imaging results under four sub-pulses

3. Adjustment of time resolution

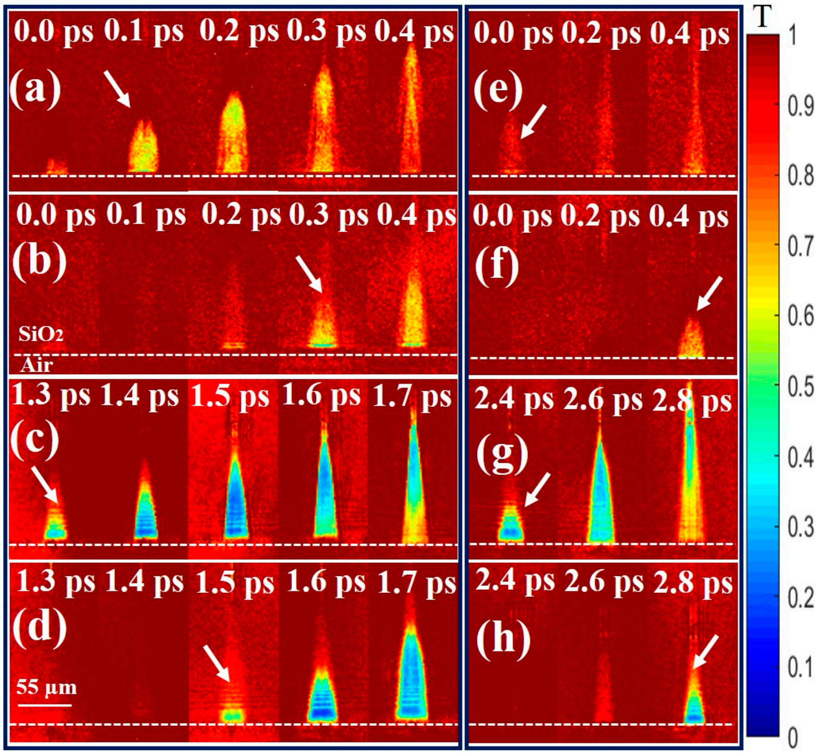

Figure S3. Different time resolutions obtained using quartz crystals of different thicknesses on (a-d) 6.4 mm and (e-h) 12.8 mm.
